# Research Priorities for the Intersection of Alcohol and HIV/AIDS in Low and Middle Income Countries: A Priority Setting Exercise

**DOI:** 10.1007/s10461-017-1921-4

**Published:** 2017-10-03

**Authors:** Sara Gordon, Mary Jane Rotheram-Borus, Sarah Skeen, Charles Perry, Kendall Bryant, Mark Tomlinson

**Affiliations:** 1 The Department of Psychology, Stellenbosch University, Private Bag X1, Matieland, 7602 South Africa; 2 Department of Psychiatry and Biobehavioral Sciences, Semel Institute, University of California at Los Angeles, 760 Westwood Plaza, Los Angeles, CA 90024 USA; 3 South African Medical Research Council, Francie van Zijl Drive, Parow Valley, Cape Town, South Africa; 4 National Institute of Alcohol Abuse and Alcoholism, 5635 Fishers Lane, Bethesda, MD 20892-7003 USA; 5 Global Center for Children and Families, Semel Institute and the Department of Psychiatry, University of California at Los Angeles, 10920 Wilshire Boulevard, Suite 350, Los Angeles, CA 90024-6521 USA

**Keywords:** HIV, Alcohol, Low and middle income countries (LMIC), Risk taking behaviors, ARV, Adherence

## Abstract

The harmful use of alcohol is a component cause for more than 200 diseases. The association between alcohol consumption, risk taking behavior and a range of infectious diseases such as HIV/AIDS is well established. The prevalence of HIV/AIDS as well as harmful alcohol use in low and middle income countries is high. Alcohol has been identified as a modifiable risk factor in the prevention and treatment of HIV/AIDS. The objective of this paper is to define research priorities for the interaction of alcohol and HIV/AIDS in low and middle income countries. The Child Health and Nutrition Research Initiative (CHNRI) priority setting methodology was applied in order to assess research priorities of the interaction of alcohol and HIV/AIDS. A group of 171 global and local experts in the field of alcohol and or HIV/AIDS related research were identified and invited to generate research questions. This resulted in 205 research questions which have been categorized and refined by senior researchers into 48 research questions to be evaluated using five criteria: answerability, effectiveness, feasibility, applicability and impact, as well as equity. A total of 59 experts participated independently in the voluntary scoring exercise (a 34% response rate). There was substantial consensus among experts on priorities for research on alcohol and HIV. These tended to break down into two categories, those focusing on better understanding the nexus between alcohol and HIV and those directed towards informing practical interventions to reduce the impact of alcohol use on HIV treatment outcomes, which replicates what Bryant (Subst Use Misuse 41:1465–1507, 2006) and Parry et al. (Addiction 108:1–2, 2012) found. Responses from experts were stratified by location in order to determine any differences between groups. On average experts in the LMIC gave higher scores than the HIC experts. Recent research has shown the causal link between alcohol consumption and the incidence of HIV/AIDS including a better understanding of the pathways through which alcohol use affects ARV adherence (and other medications to treat opportunistic infections) and CD4 counts. The results of this process clearly indicated that the important priorities for future research related to the development and assessment of interventions focusing on addressing alcohol and HIV/AIDS, addressing and exploring the impact of HIV risk and comorbid alcohol use, as well as exploring the risk and protective factors in the field of alcohol and HIV/AIDS. The findings from this priority setting exercise could guide international research agenda and make research funding more effective in addressing the research on intersection of alcohol and HIV/AIDS

The harmful use of alcohol is a component cause for more than 200 diseases and alcohol negatively effects all human organs and systems [7]. Alcohol use and subsequent co-occurring problems are highly prevalent in HIV-infected populations [12]. Alcohol consumption’s link to the disease progression of HIV was cited early in the AIDS epidemic [12]. High levels of alcohol consumption accelerate the progression of HIV [12]. Alcohol use in HIV infected individuals is associated with enhanced sexual risk-taking [13, 23], less uptake of HIV testing and care [13, 23], reduced antiretroviral treatment (ART) adherence, while persons who are HIV+ are twice as likely to not adhere to their ART regime if they are heavy drinkers [3]. In addition, alcohol affects care and outcomes at every stage of the HIV medical care cascade (i.e., diagnosis, links to medical care, engagement and retention of medical care, ART treatment and viral suppression; [1, 20, 23]).

Alcohol consumption can also affect the way antiretroviral medications are metabolised by the body [12] Furthermore, alcohol effects the interactions between drugs, hepatotoxicity and further reduced immune system functioning [13], enhances incidences of serious infections (including bacterial pneumonia and tuberculosis), negative effects on the liver including inflammation which further deteriorates the common HIV coinfection of hepatitis C [12]. Moreover excessive alcohol use and abuse effects the antioxidant system that normally protects the liver during infection [12]. Other common HIV associated coinfections in which alcohol plays a role include tuberculosis, cardiovascular disease, cancers, neurological disorders, metabolic complications, fall and injuries, other substance use and mental health disorders (see [23]).

Alcohol has been identified as a modifiable risk factor in the prevention and treatment of HIV/AIDS. However, in countries with a high burden of HIV and alcohol consumption there is insufficient acknowledgement of how interconnected they are [13, 23]. Furthermore, few HIV/AIDS prevention and treatment services, or national policies address or include alcohol as a risk factor for HIV [13]. This is a missed opportunity for prevention and treatment of alcohol and HIV/AIDS [13]. There have been calls to address the harmful linkages between alcohol and HIV through research [4, 5, 13].

Parry et al. [4] describe three research areas for alcohol and HIV/AIDS in a paper outlining the causal relationship between alcohol and HIV and the implications for policy, practice and future research. Parry et al. [4] prioritized epidemiological and etiological research to evaluate problematic alcohol consumption among newly infected HIV cases, to assess overall risk, as well as particular sub-group risk when compared to individuals who do not drink. Causal research on the correct use of condoms, concurrent sexual partners and the difference in choice of partners after heavy drinking episodes should be explored. Intervention research should test the effectiveness, feasibility and cost of interventions to reduce alcohol-related HIV risk behaviour. In addition research should compare brief interventions to more intensive interventions on alcohol reduction [4].

Following a technical consultation meeting on alcohol and infectious diseases held in Cape Town in 2012 three research areas of research should be prioritized to better understand the relationship between alcohol and infectious disease and inform policy and practice for intervention [5]. The first prioritized research on the prevalence of alcohol use, sexual HIV risk behaviours and information on treatments (including ARV adherence). The second area of research that was prioritized was randomized control trials to evaluate alcohol-focussed interventions to reduce the acquisition of HIV among HIV uninfected populations. The third prioritized research area was on the evaluation randomized control trials where interventions focussed on treatment adherence and response among persons initiating treatment for HIV and/or TB. The present research priority setting exercise aims to provide a more comprehensive and updated list of needed feasible areas for research based on a more rigorous priority setting exercise [5].

While more than $130 billion is invested in health research annually [18], however, proposals for health research far exceed the available resources [9, 10, 17]. There is therefore a need to set priorities for health research investment [16, 18]. Research priorities need to be set using sound and transparent methodologies [9, 18]. In most cases, there will never be agreement on which outcomes are correct or preferable or which decisions should be made [18]. Thus, an ethical framework that emphasises the process through which research priorities are set is needed [18, 21]. This can be accomplished through a comprehensive communication strategy which facilitates transparency [18].

The objective of this paper is to define research priorities, using the Child Health and Nutrition Research Initiative (CHNRI) priority setting methodology (described below), for the interaction of alcohol and HIV/AIDS in low and middle income countries.

## Method

### CHNRI Approach

The CHNRI research priority setting methodology, is a consensus-building tool [11, 15] that can be applied at national or global levels and for a variety of purposes addressing a single disease or a group as well as risk factors etc. [8, 10, 16, 17]. The CHNRI methodology details a list of individual questions (termed research options). These individual questions are independently scored against a pre-defined set of criteria by technical experts for each research option. The CHNRI priority setting methodology has been successfully used in a variety of global health domains (including child health, mental health and psychosocial support, developmental disabilities [6, 16, 17, 19].

## Procedure

### Establishment of a Core Group and Determining Research Criteria

The criteria against which the research options were assessed were answerability, effectiveness, feasibility, applicability and impact, and equity (See Table 1 for a list of the questions as per criterion). A group of five leading global technical experts in the area of alcohol and HIV/AIDS and formed a technical working group (TWG). The TWG consisted of MT, SS, CP and MRB. In July 2016, the TWG identified (through nominations) and invited 37 global experts to attend a meeting on the intersection of alcohol and HIV/AIDS in Durban, South Africa, funded by the National Institute of Alcoholism and Alcohol Abuse (NIAAA).

**Table 1:** The final research priority scores and ranks of the 50 research options after application of the CHNRI methodology to address the intersection of alcohol and HIV in low and middle income countries

Importance or potential impact rank (overall rank)	Research options	Answerability	Effectiveness	Feasibility	Applicability	Equity	Overall RPS	Average expert agreement (AEA)
1	What is the link between alcohol use and adherence to HIV medication?	99.1	90.4	98.2	91.4	93.4	94.5	0.92
2	What is the effectiveness of health system-based intervention that involve training facility staff to foster an enabling environment for engagement of HIV-infected patients abusing alcohol?	97.7	91.5	94.7	85.4	96	92.9	0.88
3	What is prevalence and correlates of alcohol use among HIV+ pregnant women in Africa?	98.7	91	94.9	82.8	94.3	92.3	0.89
4	How does alcohol use in HIV+ pregnant and postpartum women impact retention in care and ART adherence?	98.2	86.5	92.8	86.8	95	91.9	0.88
5	What are the barriers to getting screening and brief interventions aimed at reducing hazardous and harmful alcohol use among persons on ARV treatment routinely carried out?	94.6	88.9	95.5	87.6	92.6	91.8	0.88
6	What is the effectiveness of motivational interviewing on antiretroviral therapy adherence, virological suppression, and alcohol consumption in HIV-infected adults?	99.1	88.9	96.3	80.7	91.2	91.2	0.85
7	What are the factors associated with alcohol use among HIV positive adolescents in LMIC?	99.1	88.5	96.4	82.5	88	90.9	0.87
8	Can alcohol reduction programmes be effectively integrated into HIV prevention, treatment and care programmes?	97.7	89.1	91	87.7	88.5	90.8	0.87
9	What are the barriers to adherence in ART users who abuse alcohol in LMICs?	97.8	86.8	95	82.9	90.7	90.6	0.87
10	What is the impact of alcohol exposure on ART effectiveness?	94.8	91.3	89.8	85.7	90.7	90.5	0.84
11	What are the clinical and cost effectiveness of integrating alcohol interventions into HIV care?	96.2	86.5	93	83.8	90.8	90.1	0.85
12	To what extent do reductions in drinking in people living with HIV contribute to improved health outcomes, including both HIV and non-HIV specific outcomes?	93.3	85.8	89.6	83.2	93.3	89	0.85
13	Is there any association of alcohol use with depression and HIV disease progression among people living with HIV/AIDS?	96.5	86.3	93.1	79.6	89.6	89	0.84
14	What is the prevalence of HIV among adolescents abusing alcohol?	97.9	86	92.7	78.2	89.3	88.8	0.82
15	How can we scale up the implementation of AUD treatment programmes integrated into HIV treatment programmes in LMIC?	93.9	86.8	87.7	82.5	90.6	88.3	0.83
16	Do mobile phone brief interventions reduce alcohol use and increase adherence amongst PLWHAs?	96.4	86.1	90	81	85.3	87.8	0.82
17	Would it be feasible to use visual aids or basic cell-phone apps to give information/reminders around drug regimen and timing or amount of alcohol use?	95.5	84.5	88.5	80.7	85.8	87	0.82
18	Does alcohol abuse decrease usage of HIV testing services?	95.1	80.8	91.7	76.2	88.7	86.5	0.8
19	What is the impact of alcohol exposure on HIV disease progression?	90.9	84.3	87.7	79.7	89.1	86.3	0.79
20	What is the optimal way to screen persons who are HIV+ for hazardous and harmful alcohol use?	92.3	83.3	87.3	80.3	87.3	86.1	0.84
41	What is the impact of low risk alcohol use on immunological outcomes in HIV positive individuals?	92.1	65.5	84.2	71.3	76.1	77.8	0.72
42	To what extent can technologies be used to increase the reach of behavioral interventions to reduce drinking?	85.5	72.7	76.5	68.5	82.6	77.1	0.63
43	Which interventions make drinking venues safer in terms of likelihood of unsafe sex amongst venue patrons?	82	74.7	76.8	65.7	79.6	75.8	0.66
44	How does alcohol use in pregnancy affect liver function among pregnant women on ART?	84.6	70.8	74	64.1	79.2	74.5	0.6
45	What is the relationship between cognitive performance and alcohol consumption in people with HIV?	89	71	79.7	51	74.5	73.1	0.63
46	Can mobile phone breathalyser applications be used to assess alcohol use among PLWHAs?	90.8	68.6	73.5	56.5	68.9	71.7	0.59
47	Can Disulfiram be used for the treatment of alcohol use disorders in PLWHA?	82.2	66.7	76.7	63.1	68.6	71.5	0.58
48	Is there a safe or low-risk level of alcohol use for persons with HIV on ART?	74.3	67.7	71.1	67.2	73.4	70.7	0.59
49	What is the cost-benefit of gains from alcohol promotion and marketing versus effects on sexual behaviour and ARVs treatment for people living with HIV or at risk of HIV infection?	72.7	63.1	67.4	57.6	73.3	66.8	0.51
50	What is the effect of excessive alcohol consumption by a senior family member on adherence and compliance to ART by HIV positive dependents in the family?	78.2	58	69.7	55	72	66.6	0.55

**Table 2:** Correlation (Pearson) between mean category scores and total RPS’s across items

	Effectiveness	Feasibility	Applicability	Equity	RPS
Answerability	0.802**	0.892**	0.662**	0.635**	0.866**
Effectiveness		0.854**	0.821**	0.783**	0.946**
Feasibility			0.752**	0.664**	0.919**
Applicability				0.823**	0.910**
Equity					0.864**

** *P* < 0.001

**Table 3:** The top 5 research priority scores and ranks from experts in low and middle income countries

LMIC rank	Research question	LMIC answerability	LMIC effectiveness	LMIC feasibility	LMIC applicability	LMIC equity	LMIC RPS	LMIC AEA	Overall rank
1	What is the link between alcohol use and adherence to HIV medication?	99.2	89.7	94.9	88.8	98.3	94.2	0.91	1
2	Can alcohol reduction programmes be effectively integrated into HIV prevention, treatment and care programmes?	98.4	97.5	97.5	90.5	96.7	94.1	0.90	8
3	To what extent can technologies be used to increase the reach of behavioral interventions to reduce drinking?	94.9	91.4	96.6	90.7	96.8	94.1	0.90	23
4	Is a transdiagnostic or common elements intervention approach possible for integrating AUD treatment into HIV clinics in LMICs?	96.5	92.9	95.5	83.6	98.2	93.3	0.90	42
5	How can we scale up the implementation of AUD treatment programmes integrated into HIV treatment programmes in LMIC?	100	87.9	97.5	84.7	95.2	93.1	0.88	15

**Table 4:** The top 5 research priority scores and ranks from experts in high income countries

HIC rank	Research question	HIC answerability	HIC effectiveness	HIC feasibility	HIC applicability	HIC equity	HIC RPS	HIC AEA	Overall rank
1	What is the link between alcohol use and adherence to HIV medication?	100	93.8	99	92.6	89.6	95.0	0.93	1
2	Can alcohol reduction programmes be effectively integrated into HIV prevention, treatment and care programmes?	97.1	94	94.8	94.6	88.6	93.8	0.90	8
3	What is prevalence and correlates of alcohol use among HIV+ pregnant women in Africa?	99.1	90.9	100	85.6	89.6	93.0	0.90	3
4	What is the effectiveness of health system-based intervention that involve training facility staff to foster an enabling environment for engagement of HIV-infected patients abusing alcohol?	99	90	94	86.2	93.5	92.5	0.88	2
5	What is the role of gender and gender identity in the intersection of alcohol and HIV/AIDS epidemics?	99	89.1	93.8	87.5	93.2	92.5	0.89	21

**Fig. 1:**
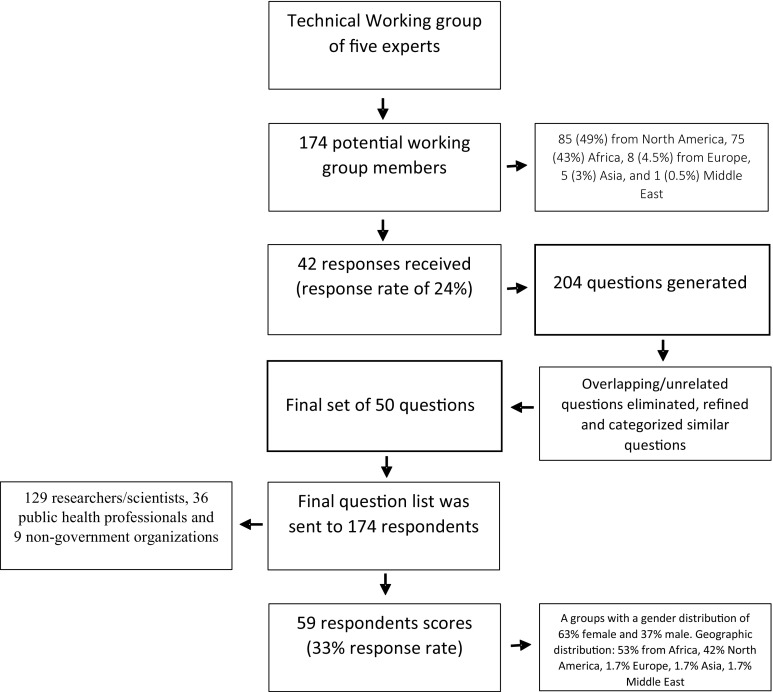
Study flowchart. Establishment of a management group and a core group

The objective of the meeting was to bring together worldwide experts on alcohol and HIV/AIDS in order to start the process of defining research priorities for alcohol and HIV in low and middle income countries. The first step of the research priority process also involved identifying the context (low and middle income countries), time-frame (next 10 years) and target population (HIV and alcohol users). This phase of the process took place during the meeting (Fig. 1).

The next phase and the second step of the research priority setting process involved generating a systematic list of research options. Each individual that attended the meeting was asked via email to identify 2–5 individuals within the field of alcohol and HIV/AIDS that could form the expanded TWG. This resulted in a group of 174 global and local experts in the field of alcohol and or HIV/AIDS related research who were invited to each generate five research questions. This resulted in 204 initial research questions. Similar questions were combined, and duplicate questions, and those not related to HIV/alcohol were deleted. This was done to reduce the number of questions to a manageable number (50 questions) and involved four rounds of consultations between four of the authors.

A final list of 50 research options to ultimately be independently evaluated using five criteria was agreed upon (Box 1). All experts who were initially approached to submit research questions, plus any additional identified experts were invited to participate in the scoring component of the research priority setting. In total 181 experts were invited to score the 50 research options sent via email. Each scoring sheet sent out was identical. This process yielded five intermediate scores (one for each criterion; ranging between 0% and 100%). Prior to the scoring, all criteria were weighed equally, as each was of equal importance. Following five follow-up emails all of the 59 returned scoring sheets were checked for errors and then scores were entered into a master calculation sheet.

**Box 1:** Questions the experts applied to each research option

**Criterion 1: Answerability** *Is the research question clear and can a study be designed to answer the research question and to reach the proposed aims of the research?* 1. Would you say that a study can be designed to answer the research question? 2. Would a study that can answer the proposed research question be granted ethical approval? **Criterion 2: Effectiveness** *Based on the best existing evidence and knowledge, would the intervention which would be developed/improved through proposed research be effective?* 1. Based on the best existing evidence and knowledge, would the intervention which would eventually benefit from the proposed research be (or become) effective? **Criterion 3: Feasibility and/or Affordability** *Is the research potentially doable in the majority of countries in the world?* 1. Is a research study to answer this question feasible? 2. Taking into account the level of difficulty with intervention delivery (e.g., the complexity of the intervention itself, the infrastructure require and human factors involved), would the proposed research be deliverable? **Criterion 4: Applicability and Impact** *Likelihood that the knowledge generated through the proposed research would be implemented and have an impact on policy and practice* 1. Do you think that the proposed research would influence policy and practice and have an impact in changing current practice? 2. Given the financial resources available to implement the intervention, would you say that its implementation would be affordable (scalable)? **Criterion 5: Equity** *Assessment of the impact of proposed research on equity* 1. Would you agree that the immediate results of the proposed research could be of help to all segments of the society, and not just the privileged ones?	

Each expert scored each research options by answering the questions per criterion about that particular option. The answers to each questions were a) no (0 points), b) yes (1 point) or c) not sure (0.5 points). In some instances experts may not have felt informed enough to answer a research option. In these cases answers were left blank. The methodology deals with missing answers as it is not expects that each expert has all the necessary knowledge on each possible research option to score it against the criterion [9]. Moreover, according to the wisdom of crowds theory individuals in the rating process have the chance to express a judgement (i.e., score research items that they prioritize) and this judgement is treated equally. Naturally, these judgements include personal biases but these tend to be cancelled out or diluted [14, 19].

Intermediate research priority scores are calculated by summing all the informed answers (i.e., “1”, “0.5” or “0”). This sum was divided by the number of received informed answers (blanks are left out of the numerator and the denominator). This results in research priority scores (RPS) between 0 and 100%. The RPS represents a score of how much the experts believes that the research options would satisfy the priority setting criteria (see Box 1; answerability, effectiveness, feasibility, applicability and impact, or equity; [9, 17]. RPS’s and average expert agreement scores were calculated for each research option. The more experts who agree to participate in the scoring, the more reliable the outcomes of the research priority setting will be [9, 17].

In terms of reliability or agreement, an average expert agreement statistic was generated for each research option across the five criteria. The missing (or undecided; “0”) responses meant that a Fleiss Kappa statistic to assess agreement was not appropriate (see [17]. This is in accordance with previous research priority exercises that used the CHNRI methodology [22]. With a large number of scorers and few scoring options it is possible to create a chance Fleiss Kappa [22]. The AEA statistic is an average proportion of scorers that agreed on the eight questions asked (see Box 1). Although the AEA does not give an indication of statistical significance, it was assumed that funders and or policy makers would find it more useful as it creates a general overview of the agreement between experts [22]. The AEA was calculated for each research option as follows:

$${\text{AEA}} = \frac{1}{8} \times \sum\limits_{{{\text{q}} = 1}}^{8} {\frac{{{\text{N }}({\text{scorers}}\,{\text{who}}\,{\text{provided}}\,{\text{the}}\,{\text{most}}\,{\text{frequent}}\,{\text{response}})}}{{{\text{N}}\,({\text{scorers}})}}}$$

In order to compare responses a comparative analysis of scores was undertaken. Responses were stratified by location of respondents/participants (low and middle income countries and high income countries). A Spearman’s Rho correlation coefficient was calculated to determine the correlation of research questions’ ranks between these groups. Spearman’s Rho correlation determines the degree of correlation between two sets of ranked research questions. A correlation coefficient of 1 indicated a high, positive association between two ranked sets; correlation coefficient of −1 indicate a high negative association between two ranked sets, and a correlation coefficient of 0 indicates no association.

## Results

A total of 59 experts participated independently in the voluntary scoring exercise (a 34% response rate). However, we received scores from 27 of the 42 respondents who submitted research questions (a response rate of 64%). Scoring took place over three months in 17 (February–April). The respondents consisted of 41 researchers/scientists, 15 from the field of public health and 3 individuals the non-governmental organizations. The gender distribution was 63% female and 37% male. While the geographic distribution was 53% from Africa, 42% from USA, 1.7% from United Kingdom, 1.7% from Asia, 1.7% from the Middle East (53% from low and middle income countries (LMICs) and 47% from high income countries (HICs).

The final results of the scoring process (top 20 and bottom 10) are shown in Table 1. The scored research options are ranked by their final RPS multiplied by 100, which results in scores between 0 and 100. The final RPS’s for the 50 research options ranged from 66.6/100 to 94.5/100. This range shows significant variation, indicating that the methodology has the power to discriminate among many competing research options using a single conceptual framework with eight questions.

For the top twenty research options, the average expert agreement was between 79/100 and 92/100. This means that between 8 and 9 experts out of ten gave the same score to each of the research options for the top twenty research options. This level of agreement is higher than expected from random assignment of scores of zero or one, because an undecided answer is also allowed. This demonstrated that the experts largely agreed on the RPS’s (for all criteria) overall but did not agree on the scores for the research options at the bottom of the ranking list, for which the AEA scores ranged from 50/100 to 60/100 (see also [17].

The most prevalent question theme deals with interventions in the field of alcohol and HIV/AIDS. Nine of the research questions in the top twenty fall into the research question theme of interventions on the intersection of alcohol and HIV/AIDS (i.e., intervention impact, intervention development or cost-effectiveness of interventions for alcohol and HIV/AIDS). Most of these research options were scored in the top ten, as five of these research options were ranked in the top ten. Research options in this theme all scored very highly on the answerability criteria (scores ranged from 92.3/100 to 99.1/100) as well as others.

Another prominent question theme deals with the impact of HIV risk and comorbid alcohol use. Four research questions in the top twenty fall into this research question theme (including the highest ranked research option). Research options in this theme also all scored highly on the answerability criteria (scores ranged from 93.3/100 to 99.1/100). These question also scored highly on the feasibility criteria (scores ranged from 89.6/100 to 98.2/100) as well as on the equity criteria (scores ranged from 88.7/100 to 95/100).

The question theme of risk and protective factors for alcohol and HIV was also prominent with four questions in the top twenty scored research options. These four questions also scored highly on answerability (with scores ranging from 90.9/100 to 99.1/100). These research questions also scored highly on equity (88/100–93.3/100).

The scoring of the 50 research options resulting in the ranking of research options based on the likelihood that they would be answerable, effective, feasible and or affordable, applicable or have impact or have in impact on equity. An overall RPS is computed as the mean of each criterion score. Mean scores for each criterion were highly inter-correlated Table 2, while Table 1 shows the mean RPS scores for each research criteria.

The highest scoring research option was “*What is the link between alcohol use and adherence to HIV medication?*” This question scored very highly on answerability (99.1/100), effectiveness (90.4/100) and was considered feasible (98.2/100), applicable (91.4/100) and equitable (93.4/100). This question was accepted by the expert group to be the most likely to generate original knowledge, with a total RPS of 94.5/100. Just over nine out of ten experts agreed on scores for this research option (AEA 92/100). This was also the highest AEA score.

The second highest scoring research option “*What is the effectiveness of health system*-*based intervention that involve training facility staff to foster an enabling environment for engagement of HIV*-*infected patients abusing alcohol?*” This question highly on all five criteria (answerability: 97.7/100; effectiveness: 91.5/100; feasibility: 94.7/100; applicability 85.4/100 and equity: 92.9/100). The total RPS for this research option was 92.9/100 and was agreed on by experts that it would too generate original knowledge as just under 9 out of ten experts agreed on scores for this research option (AEA 88/100).

The third highest scoring research option is an epidemiological question asking about the prevalence on alcohol use among HIV+ pregnant women in Africa *(“What is prevalence and correlates of alcohol use among HIV*+ *pregnant women in Africa?”).* Scores on all five criteria were equally as high for this research option (answerability: 98.7/100; effectiveness: 91/100; feasibility: 94.9/100; applicability 82.8/100 and equity: 94.3/100) with a total RPS of 92.3/100. Experts had slightly higher agreement on scores for this research (AEA 89/100) compared to the second highest rated research option. The applicability scores for the top three highest rated research options were the lowest scores for each research item.

The lowest scoring research option in the present priority setting exercise concerned the effects of a senior member of a family drinking excessively (*“What is the effect of excessive alcohol consumption by a senior family member on adherence and compliance to ART by HIV positive dependents in the family?”).* The expert group rated this research option fairly low on effectiveness (58/100) and applicability (55/100), but scores for answerability (78.2/100) feasibility (69.7/100) and equity (72/100) were higher than expected, with a total RPS of 66.6/100. Moreover, just over five out of then experts agreed on the scores for this research option (AEA 54.6/100).

There were only three other questions which had scores for any criteria below 60%. *“What is the cost-benefit of gains from alcohol promotion* *and marketing versus effects on sexual behaviour and ARVs treatment for people living with HIV or at risk of HIV infection?”* scored fairly high on answerability (72.7/100), equity (73.3/100) while scores for effectiveness, feasibility and applicability were low with a total RPS of 66.8/100. This question has the lowest AEA score of 51/100, where only half of the experts could agree on a score for this research option. The third lowest score was given to *“Can mobile phone breathalyser applications be used to assess alcohol use among PLWHAs?”* on applicability (57.6/100), with a total RPS of 71.7/100. This research option also scored the third lowest on AEA (59.4/100).

*“What is the relationship between cognitive performance and alcohol consumption in people with HIV?”* scored highly on answerability (89/100), effectiveness (71/100), feasibility (79.7/100) and equity (74.5/100) but was scored the lowest (of any criteria for any question) on applicability (51/100), with a total RPS of 73.1/100. This question has the second lowest AEA score of 55/100, where just over five experts out of ten could agree on the scores for this research option.

### Low and Middle Income Country (LMIC) Scores Versus High Income Country (HIC) Scores

Responses from experts were stratified by location in order to determine any differences between groups. The LMIC expert group RPS (mean 84.49; range 67.53–94.11) were slightly higher than their HIC expert group counterparts (mean 81.87; range 59.81–94.98) meaning that on average experts in the LMIC gave higher scores than the HIC experts. The experts in the HIC gave scores with a slightly larger range when compared to the LMIC. The Spearman’s Rho correlation of the research questions ranks between LMIC experts and HIC experts was moderately weak to positive, though statistically significant (Spearman’s r = 0.466, *p* < 0.01). While there were discrepancies between research questions prioritized by the LMIC experts versus the HIC experts, both groups of agreed on the first overall research option and scored it highly. Both groups also agreed on the second research question (rated eighth overall; *“Can alcohol reduction programmes be effectively integrated into HIV prevention, treatment and care programmes?”*) (Tables 3, 4).

## Discussion

There was substantial consensus among experts on priorities for research on alcohol and HIV. These tended to break down into two categories, those focusing on better understanding the nexus between alcohol and HIV and those directed towards informing practical interventions to reduce the impact of alcohol use on HIV treatment outcomes. This replicates what [5] found following technical consultation on alcohol and HIV held in Cape Town in 2012. The results of the present priority setting exercise are further replicated in [2] where research on alcohol use in HIV+ pregnant women was prioritized. In this exercise research in this areas was the third highest rated research item (overall and by the HIC experts). Research in this area is international focus in under-resourced settings in Africa [2] but experts in the LMIC did not rate this research item in the top 5 research items. In order to substantially address the significantly problems stemming from intersection of alcohol and HIV/AIDS research funding should focus on interventions that focus on alcohol and HIV/AIDS (i.e., intervention impact, intervention development or cost-effectiveness of interventions for alcohol and HIV/AIDS).

Although, the highest rated research option relates to the impact that alcohol has on HIV/AIDS. More specifically, how alcohol effects adherence to ARV medication. This research option scored particularly high on all five categories (i.e., answerability, effectiveness, feasibility, applicability and equity). This research option also had the highest level of consensus amongst experts. Where nine out of ten worldwide experts in the field of alcohol and HIV/AIDS agreed on research priority scores for this research option (AEA for this research option was 92/100).

As in many other priority setting exercises the difference in total RPS’s is less than four percentage points. Moreover the difference in scores between the top ranked research option and the research option ranked twentieth is only 8.4 percentage points. These score are also all fairly high in comparison to other research priority setting exercises. This demonstrates that research options within this field are highly contestable and are all considered by these experts to be a priority within this field. Moreover, this is the opinion of worldwide experts within the field of alcohol and HIV/AIDS.

However, the biggest difference (1.6 percentage points) between research options is between the first and second research option. This attests to the recognition that alcohol affects how PLWHA adhere to their ARV medication and the importance that experts placed on understanding and exploring how alcohol effect ARV medication adherence. Although research has shown that alcohol reduces ARV adherence [3, 13, 23] this group of experts still rated this research option as the most important research priority. Indicating that more research still needs to be done in this area.

A significant feature of this priority setting exercise was the relatively high final RPS’s (as well as the individual scores in the five criteria’s) of the 50 research options when compared to other research priority setting exercises using the CHNRI methodology (see [17, 19, 22]. It is difficult to ascertain the cause of the high scores in the current exercise. However, it could be due to the shared optimism and sense of urgency amongst the group of experts who participated in this priority setting exercise. This is in accordance with Schneider et al. [13] who concluded in their paper entitled: *Alcohol consumption and HIV/AIDS: the neglected interface* that the harmful linkages between alcohol and HIV need to urgently be addressed within research.

Nevertheless, it could also be due the choice of criteria that facilitated the high scores found within this exercise. The answerability criteria for the top twenty research options range from 92.3/100 to 99.1/100 (the range for the 50 research options for answerability was 72.7/100–99.1/100). These high scores indicate the shared optimism of this expert group that all the research questions are answerable.

The CHNRI methodology to research priority setting generates specific outcomes with priority scores [17]. These scores provide insight on the risk associated with each specific research option [17]. Although this research priority setting methodology should only be used as a guide to which research options can be judged. Thus, final scores should be seen as a guide to investment as funding decisions will always be directed by funding and donor priorities. For example, *“Which skill helps primary health workers to improve ARV treatment adherence among alcohol abuse clients?”* This question was ranked 27th (out of 50) but an answer to this question could provide very useful data for the treatment of PLWHA in LMIC.

Although a large number of experts generate research options that were ultimately rated, it is unattainable, in such an exercise to create a comprehensive list of research options. As a result, both the research options and the rating generated are likely to replicate biases the identification, sampling and participations of experts. In addition, participants not fluent in English and experts who have difficulties in simultaneously working with complex information would have limited participation in such an exercise. It is conceivable that the scores of exerts who responded could be systematically different from those who did not respond [22]. The low response rate was, we believe, a result of the time consuming nature of the exercise. Our findings might also be biased towards the views of researchers and scientists. It is possible that had more participants from other sectors (e.g. health care workers working at clinic level) been included that the responses would have differed.

## Conclusion

The main strength of this priority setting exercise is that (1) a proven methodology was undertaken [9, 16, 19], (2) a significant number of experts with an acceptable gender balance and geographical spread participated in the exercise. Recent research has shown the causal link between alcohol consumption and the incidence of HIV/AIDS including a better understanding of the pathways through which alcohol use affects ARV adherence (and other medications to treat opportunistic infections) and CD4 counts. We believe that these priorities have the potential to inform decision making in the area of for funding organizations and policy makers. Future research should look at including a broader grouping of experts from a wide range of sectors. In addition experts from countries like India and Russia should be included.

Despite study limitations, we were successful in producing research options from a significant number of global experts. In addition, this study demonstrates a need to invest in research that focuses on attempting to develop or assess interventions that concentrate on the addressing the intersection of alcohol and HIV/AIDS, both continuing to seek to better understand the nexus between alcohol and HIV while at the same time testing suitable interventions aimed at reducing alcohol’s impact on the incidence of HIV and HIV disease progression. This research priority setting exercise provides a significant contribution to establishing research priorities in the field of alcohol and HIV/AIDS, as the information was gathered from a group of experts, all relevant experience. As far as can be ascertained this is the first priority setting exercise within this research domain. The findings from this priority setting exercise could guide international research agenda and make research funding more effective in addressing the research on intersection of alcohol and HIV/AIDS. The key research question to be answered according to the results of this study are around the development and assessment of interventions focusing on addressing alcohol and HIV/AIDS, addressing and exploring the impact of HIV risk and comorbid alcohol use, as well as exploring the risk and protective factors in the field of alcohol and HIV/AIDS.
